# Evaluation of spreading vasomotor responses in isolated human cerebral arteries

**DOI:** 10.1016/j.mex.2026.104086

**Published:** 2026-08-02

**Authors:** Donald G. Welsh, Suzanne E. Brett, Melfort R. Boulton, Anil Zechariah

**Affiliations:** a Robarts Research Institute and the Department of Physiology and Pharmacology, University of Western Ontario, London, Ontario, Canada; b Department of Neurology, University of Western Ontario, London, Ontario, Canada; c Division of Biomedical Sciences, Faculty of Medicine, Memorial University of Newfoundland, St. John’s, NL, Canada

**Keywords:** Cell-cell communication, Myography, Conduction assay, Conducted vasomotor responses, Vasoconstriction

## Abstract

•The protocol outlines an approach to quantify electrical communication in isolated human cerebral arteries, facilitating insights into human cerebrovascular physiology and regulation of cerebral blood flow.

Optimal neural activity depends on adequate brain perfusion, requiring precise coordination between neuronal metabolic demand and vascular responses. This coupling depends on intercellular communication within the vasculature, which remains poorly characterized in the human cerebral circulation. In this regard, we present the “conduction protocol,” a functional method to assess electrical signal propagation along human cerebral arteries. Cerebral surface arteries (∼220 µm diameter, ∼2000 µm length) were isolated from resected human brain tissue, cannulated, pressurized in a myography chamber, and superfused with physiological salt solution to maintain viability. A localized high concentration of potassium chloride was applied to the arterial wall using a micropipette, and vasoconstrictive responses were measured at the application site and at distal locations up to 1800 µm. A strong constrictive response was consistently observed across all measured sites, with a gradual decay in magnitude as the signal propagated along the vessel. This protocol validates conducted vasomotor responses in human cerebral arteries and provides a practical and accessible approach to assess vascular intercellular communication and cerebrovascular responses in the human cerebral circulation.

## Graphical abstract

**Figure:**
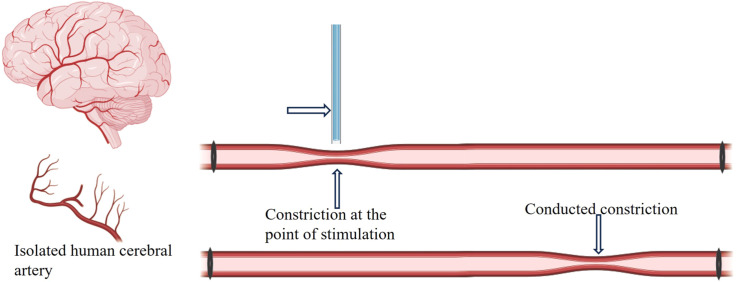
The graphical abstract was created in BioRender. Zechariah, A. (2026) https://BioRender.com/6zkpcqd.

**Specifications table**

**Table:** 

**Subject area**	Medicine and Dentistry
**More specific subject area**	Cerebrovascular Physiology
**Name of your protocol**	Vasomotor conduction assay
**Reagents/tools**	Not applicable
**Experimental design**	Human cerebral arteries were isolated, cannulated and pressurized on a pressure myograph. Vascular stimulants were exposed to a small region of the cannulated blood vessel. Conducted vasomotor responses were then quantified at sites, up to 1800 µm from the point of application.
**Trial registration**	Not applicable
**Ethics**	All procedures involving human tissue have been approved by the Human Research Ethics Board at the University of Western Ontario, London, Ontario, Canada.
**Value of the Protocol**	1. This protocol enables direct measurement of conducted vasomotor responses in human cerebral arteries. 2. This protocol offers a practical reproducible method to quantify vascular intercellular communications in the human brain. 3. This protocol further provides a platform for examining cerebrovascular dysfunction directly in human tissue.

## Background

The maintenance of normal brain function depends on the precise coupling of neural activity with adequate blood flow delivery. To achieve significant changes in cerebral blood flow, locally generated signals arising from neural activity must initiate vasomotor responses that propagate upstream to resistance vessels that reside on the brain surface. These coordinated responses ensure that regional metabolic demands are met efficiently within the cerebral circulation.

The ability of vasomotor responses to spread along arteries and across branch points was first described by August Krogh over a century ago in the frog hindlimb [1]. Spreading vascular responses are independent of neural innervation or hemodynamic changes, and result from the longitudinal movement of electrical charge along vascular cells [2,3]. This process of intercellular communication is enabled by gap junctions, intercellular pores that couple neighboring smooth muscle cells, adjacent endothelial cells, and the two cell layers. The precise pattern of communication entails the spread of charge, first along the endothelial cell layer and followed by its distribution to the overlying smooth muscle via myoendothelial gap junctions [[4], [5], [6]]. Functionally, electrical signals preferentially propagate along the highly coupled endothelial layer before being transmitted to the overlying smooth muscle cells through the comparatively less coupled myoendothelial gap junctions, leading to coordination of vasomotor activity along the blood vessel [[4], [5], [6]]. The dependence of conducted responses on an intact endothelium was directly evaluated in our previous study (Zechariah et al., 2020 [7]) using the same cerebral artery preparation and KCl stimulus-based conduction paradigm. In that work, we demonstrated that passing an air bubble through the lumen selectively disrupted the endothelial layer and abolished conducted vasomotor responses beyond the stimulation site, while the local KCl‑evoked constriction at the site of stimulation remained intact [7]. Furthermore, in our study (Zechariah et al., 2020), genetic ablation of endothelial gap junction forming protein connexin 40 (Cx40), diminished, but did not eliminate conducted vasomotor responses and attenuated the longitudinal spread of membrane depolarization along the cannulated artery, consistent with impaired gap junctional communication [7]. Our findings were also consistent with prior work in arteries and arterioles in which selective endothelial damage prevented conducted vasodilation and hyperpolarization from propagating beyond the denuded segment, whereas damage confined to the smooth muscle cell layer displays the opposite pattern [[8], [9], [10]].

The conduction assay first described by Segal and Duling [11], is a functional means of assessing cell-to-cell communication in resistance arteries. On a defined length of artery, vasoactive agents are discretely applied to a small portion of the artery via micropipette and vasomotor and/or V_M_ responses are measured at sites, on and distal, from the point of application. By monitoring the distance over which responses spread under distinct experimental conditions, experimenters can deduce the mechanistic basis of the vascular response and how disease can alter this critical process. While conduction assay is considered the gold standard for evaluating cell-cell communication, this method of assessment has not been adapted and extended to human arteries including that from the brain.

Here, we describe the utility of a conduction protocol in cerebral arteries isolated from resected human brain tissue. We outline the isolation, cannulation, and pressurization of human cerebral arteries, induce a local vessel constriction, and monitor the conducted response along the vessel. We also discuss the methodological implications and highlight how documenting conduction may advance understanding of electrical communication between vascular cells in a translational setting.

## Description of protocol

All procedures involving human tissue have been approved by the Human Research Ethics Board at the University of Western Ontario, London, Ontario, Canada.

### Tissue acquisition and vessel isolation

As reported in our study (Zechariah et al., 2020 [7]), a total of 11 cerebral arteries were collected from 6 patients (3 males and 3 females; 27–59 years of age) undergoing epileptic resection surgery out of which 3 were used for conduction assay [7]. All samples were obtained after written, informed consent from patients. Resected brain sections were collected in a sealed container with saline solution on ice and were transported to the lab in an insulated carrier bag. The collected brain tissue was maintained in cold phosphate buffer solution (PBS; pH7.0) containing, in mM, 138 NaCl, 3 KCl, 10 Na_2_HPO_4_, 2 KH_2_PO_4_, 5 glucose, 0.1 CaCl_2_, and 0.1 MgSO_4_) [7,12].

Pressure myography generally requires an unbranched, straight segment that is long enough to accommodate both cannulation pipettes and to leave an intact central region for diameter tracking. As our conduction assay measured conducted responses up to 1800 µm from the point of stimulation, we used isolated arteries that were at least 2000 µm in length with a minimum number of branches. In our experience arteries which are in the 150–250 µm diameter range typically provided relatively long arterial segments with minimal branching. When branches were unavoidable, they were closed completely by pinching with forceps without damaging the main vessel segment. Under a dissecting microscope, surface arterioles (> 2 mm in length; 150–250 µm in diameter) were carefully isolated by removing surrounding connective tissue and was dissected out of the arterial network.

Freshly isolated cerebral arteries cannulated within one hour of collection were used for the conduction assay. All three isolated human cerebral arteries were successfully cannulated and pressurized, and each exhibited robust conducted responses to the stimulus in our experiments. Given the dependence of conducted vasomotor responses on an intact endothelial cell layer, isolated arteries were handled with particular care to preserve endothelial integrity. Our inclusion criteria strictly excluded any vessels with visible damage incurred during isolation.

### Solutions and setup

**Fig. 1:**
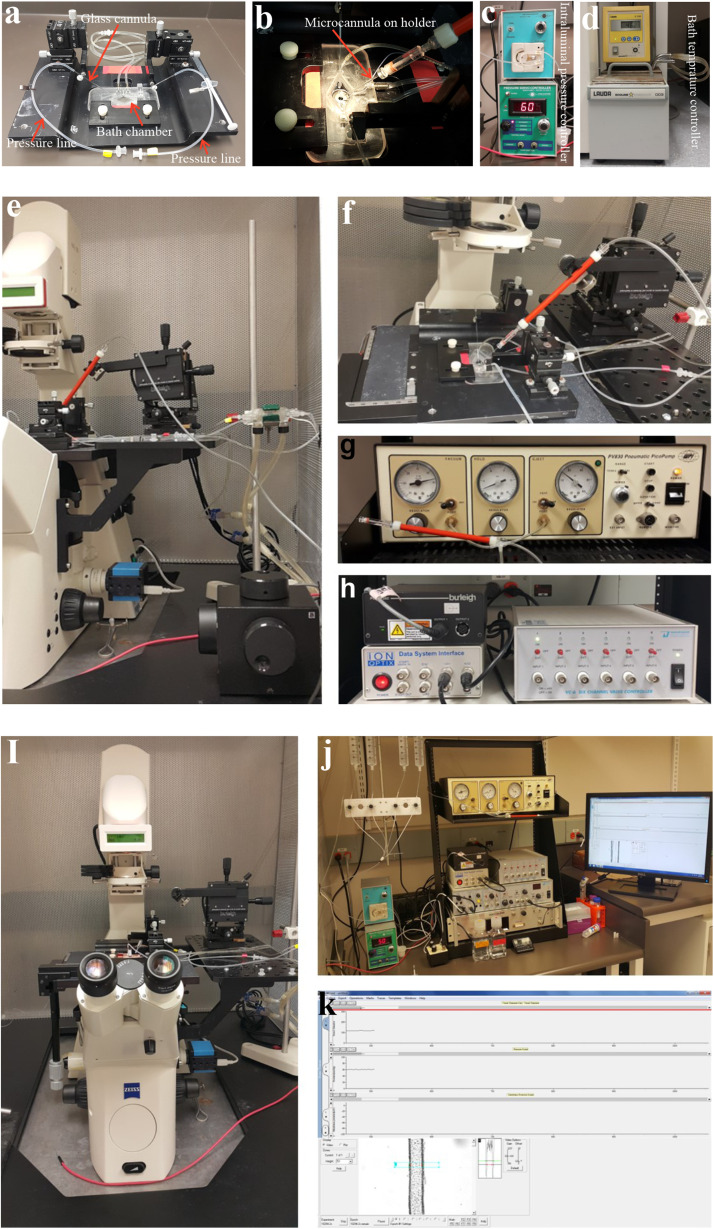
Experimental setup for assessing conducted vasomotor responses in isolated human cerebral arteries. (a-d) Microscope platform with vessel perfusion bath chamber is setup with glass cannulas, pressure lines, fluid inflow/outflow lines and is connected to external pressure and temperature control devices. (e-g) Microcannulas for stimulus delivery are mounted in a micropipette holder and connected to a pneumatic picopump system. (h-j) Micropipette manipulation and flow to perfusion chamber are regulated by the associated control boxes. (k) Changes in internal and external vessel diameter are recorded using an Ion Optix data acquisition system attached to the microscope through a cooled camera device.
c. Vessel perfusion bath chamber: The custom perfusion bath chamber (Fig. 1b) is constructed from translucent acrylic plastic and milled to include an enclosed circulating water bath chamber that helps maintain bath temperature (approximately 36.5 °C). Three additional ports are also milled to accommodate the in-flow tubing, out-flow tubing, and a micro-temperature probe. The bath chamber is designed to fit securely within the microscope platform shown in Fig. 1a.
d. External pressure control: Intraluminal pressure is controlled externally using a Living Systems PS200 pressure servo unit (Fig. 1c). Tubing connected to the pressure sensitive probe and the pressure servo unit is connected to one of the vessel cannula and the clamp is removed. The second clamp on the opposing cannula must remain in place to preserve the closed pressure system. Perfusion is initiated at 15 mmHg line pressure and then raised to 50 mmHg for experimental recordings.
e. Temperature control. An external heat source is required to warm both the perfusion chamber and the PSS solution before it enters the bath chamber. A small circulating water bath (Lauda 003E100), in combination with line splitters and connectors (Fig. 1d and e), can be used for both functions. A temperature probe placed in the perfusion chamber is used to monitor the thermal environment of the vessel, and the ideal temperature for cerebral vessels is approximately 36.5 °C.
f. Cannula and tubing: Borosilicate glass tubes, outer diameter 1.2 mm; inner diameter 0.69 mm; length 15 cm) were pulled on a micropipette puller (P-97; Sutter Instruments Co.) with heat set at 650, pull at 160, velocity at 60 and time at 70 to create two cannulas of approximately equal length. The tips were then broken under a dissecting microscope to produce a fine outer diameter of <30 μm and fire-polished with a micro-forge (MF-830; Narishige Scientific Instrument, Tokyo, Japan) to smooth the broken edges. Silastic tubing (Dow Corning, outer diameter 2.16 mm; inner diameter 1.02 mm) was connected to each cannula, prefilled with PSS+Ca^2+^, and subsequently clamped to maintain fluid within the cannulae (Fig. 1a).
g. Vessel setup: A segment of arteriole, approximately 2 mm in length, previously dissected from the brain tissue, was transferred to PSS-Ca^2+^ solution in the acrylic chamber and one end was tied to the cannula using a single thread strand. The tubing was secured with a micro-clamp (Schwartz Micro Serrefines; Fine Science Tools, Canada). The opposite end of the vessel was mounted on the second cannula and tied securely with a second thread. The cannula tips were positioned a few microns above the chamber bottom to allow complete perfusion of solution around the vessel. Depending on the experimental protocol, the lines were prefilled with either PSS-Ca^2+^ or PSS+Ca^2+^. A minimum of three lines were prepared: one line of PSS with no added Ca^2+^ and two lines with Ca^2+^ PSS. The rig was prepared for experimentation by flushing PSS-Ca^2+^ and PSS+Ca^2+^ solutions through the tubing using a Warner pinch clamp valve system.

a. Perfusion solutions: Physiological saline solution (PSS) was prepared fresh at pH 7.4, equilibrated with 5% CO_2_ in air and contained, in mM 119 NaCl, 4.7 KCl, 20 NaHCO_3_, 1.1 KH_2_PO_4_, 1.2 MgSO_4_, 1.6 CaCl_2_, and 10 glucose [13]. Two separate PSS solutions were prepared: calcium-free PSS (PSS-Ca^2+^ (PSS) and PSS with added calcium (PSS+Ca^2+^).
b. Microscope platform: The microscope platform (Fig. 1a) was custom designed to meet the requirements of the microscope. Constructed from anodized aluminum, the platform provides 360-degree support for cannulated vessels using fixed micro-manipulators mounted on a Newport M-MT-XYZ compact metric dovetail linear stage, allowing three-dimensional movement in the front/back, side/side, and up/down directions.

### Vessel viability test

Vessel viability was assessed at baseline by a brief pulse exposure to 60 mM KCl, which induces a marked constriction and rapid recovery in vessels not damaged during the dissection procedure.

### Focal stimulation of the artery

Focal stimulation of pressurized arteries was achieved by pressure ejection of 500 mM KCl through a microcannula at 30 psi for 10 s on to a discrete portion of the artery [7,14].

The microcannulas were prepared from borosilicate glass capillary tubes (Sutter; outer diameter 1.2 mm; inner diameter 0.69 mm; length 10 cm) pulled on a micropipette puller with the heat set at 615, pull at 80, velocity at 60, and time at 200. The micropipette was mounted in a pipette holder attached to a micromanipulator (Burleigh PCS-5000; Fig. 1e and f), with control tube (Fig. 1e), and connected to a pneumatic picopump system (World Precision Instruments, PV830; Fig. 1g and h).

After focal stimulation, the spread of conducted response was traced in the direction opposite to, and upstream from, perfusion flow.

### Data acquisition

Once the vessel platform is positioned on the microscope (Fig. 1i), the pressure line is connected to the cannulated vessel. The hot-water circulating lines, temperature probe and the inflow and out flow lines are then inserted into the perfusion chamber (Fig. 1e, f, j). The microscope is connected to the data acquisition system using a cooled camera device (Fig. 1i). Real-time data are acquired using a diameter recording system and software (IonOptix; Fig. 1h) capable of tracking changes in both luminal and abluminal vessel diameter in cannulated blood vessels (Fig. 1k).

## Protocol validation

### Representative results

**Fig. 2:**
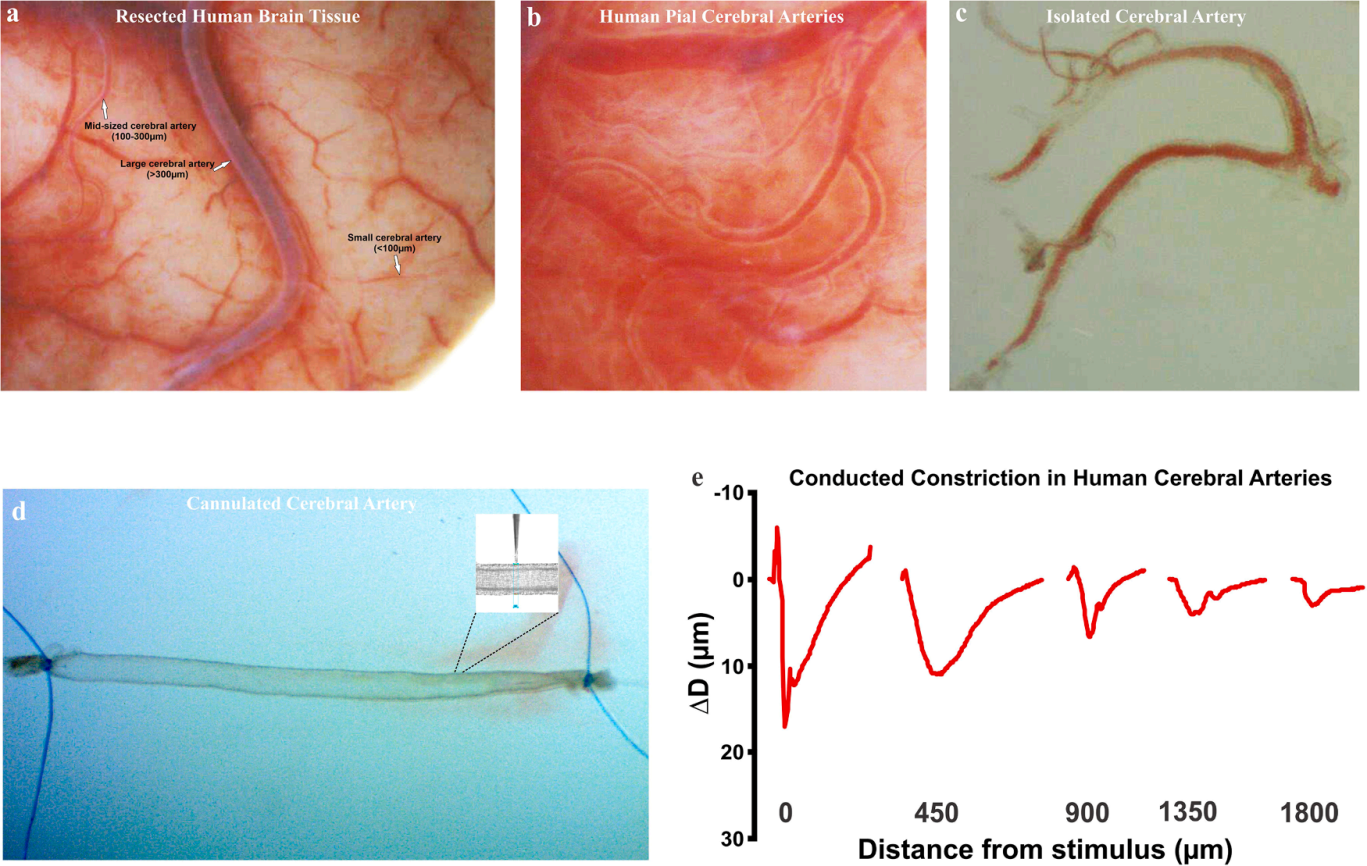
Isolation, cannulation and assessment of conducted vascular responses in human cerebral arteries. (a-c) Human cerebral arteries (> 2 mm in length; 150–250 µm in diameter) were isolated and separated carefully to minimize vessel damage. (d) The arteries were then cannulated, pressurized, and exposed to focal stimulation via micropipette injection of the stimulus-inducing compound. (e) Following KCl application, the conducted constriction response and its electrotonic decay were quantified at sites located 0, 450, 900, 1350 and 1800 µm from the point of stimulation.

Arteries isolated from human brain tissue (Fig. 2a-c) were viable, as evidenced by a robust constriction in response to 60 mM KCl in the bath followed by recovery to baseline diameter after washout. Focal application of KCl via micropipette to a small segment of the cannulated artery (Fig. 2d) elicited a pronounced constriction at the site of stimulation (Fig. 2e). This response then propagated along the arterial wall and was detected at distances of up to 1800 µm distal to the site of application (Fig. 2e). The conducted response decayed with distance along the artery, consistent with the electrotonic properties of smooth muscle and endothelial cells. The conducted responses observed in human cerebral arteries extended over distances comparable to those reported in animal arteries isolated from skeletal muscle 15, mesenteric [16] and cerebral [17] tissues. The presence of a conducted response confirms the presence of robust electrical communication in the human cerebral circulation.

## Limitations, pitfalls and mitigation recommendations

Working with human cerebral arteries introduces distinct challenges compared with blood vessels isolated from animal models, including greater variability in patient age, comorbidities, and perioperative exposure to medications. Isolated human cerebral arteries also tend to be more tortuous, which complicates dissection and cannulation and makes it more difficult to maintain a stable myogenic tone. To address these issues, our protocol incorporates strict inclusion criteria, including exclusion of samples from patients older than 60 years of age and use of only those tissues that meet a stringent maximum interval of 60 min from collection to artery isolation.

Mechanical damage during vessel dissection and isolation due to stretching, crushing or drying could lead to loss of myogenic tone and blunted vasomotor responses. To minimize this risk, all dissections should be performed in cold physiological saline solution with the vessel handled only by the adventitia and kept fully submerged at all times. Air bubbles introduced into the lumen can denude the endothelium and irreversibly impair function; hence cannulation pipettes and tubing must be prefilled carefully, vessels mounted while completely submerged, and any preparation through which a visible air bubble has passed should be discarded. Functionally, arteries that fail to develop stable myogenic tone or that exhibit weak constriction to a standardized 60 mmol/L KCl pulse are unsuitable for conduction measurements and should be excluded. Finally, temperature fluctuations, chamber vibration, or measurement regions close to cannulation sites can introduce motion artefacts and compromise automated edge detection.

In light of these considerations, we developed a stepwise quality-control pipeline that evaluates (1) successful, non-leaking cannulation of isolated arteries, (2) stable maintenance of intraluminal pressure, and (3) robust contractile responses to high-KCl depolarization in the bath solution. Implementation of these stringent criteria and workflow resulted in the consistent demonstration of conducted vasomotor responses across all human cerebral arteries subjected to the conduction assay, in our study.

The present protocol focuses on human brain surface artery segments and therefore cannot assess conducted responses across branching networks, including penetrating arterioles and capillaries which are important components of the neurovascular signaling system. Future adaptations could extend this approach to incorporate these additional microvascular segments within the cerebrovascular signaling network.

## Related research article or a published article

Zechariah, A. et al. Intercellular Conduction Optimizes Arterial Network Function and Conserves Blood Flow Homeostasis During Cerebrovascular Challenges. *Arterioscler. Thromb. Vasc. Biol.* **40**, 733–750 (2020). *https://pmc.ncbi.nlm.nih.gov/articles/PMC7058668/*

## CRediT authorship contribution statement

**Donald G. Welsh:** Conceptualization, Methodology, Supervision, Writing – review & editing. **Suzanne E. Brett:** Methodology, Validation. **Melfort R. Boulton:** Conceptualization, Writing – review & editing. **Anil Zechariah:** Conceptualization, Investigation, Methodology, Writing – review & editing.

## Declaration of competing interest

The authors declare that they have no known competing financial interests or personal relationships that could have appeared to influence the work reported in this paper.

## Data Availability

Data will be made available on request.
